# COVID-19: Um Novo Desafio para a Cardiopatia na Gravidez

**DOI:** 10.36660/abc.20200511

**Published:** 2020-07-28

**Authors:** Walkiria Samuel Avila, Regina Coeli de Carvalho

**Affiliations:** 1 Universidade de São Paulo Instituto do Coração São PauloSP Brasil Universidade de São Paulo Instituto do Coração, São Paulo, SP - Brasil; 2 Hospital Geral de Fortaleza Secretaria de Saúde do Estado do Ceará FortalezaCE Brasil Hospital Geral de Fortaleza, Secretaria de Saúde do Estado do Ceará (SESA), Fortaleza, CE - Brasil

**Keywords:** Coronavírus, COVID-19, Pandemia, Gravidez, Gravidez de Alto Risco, Síndrome Respiratória Aguda Grave, Pneumonia, Hipertensão/prevenção e controle, Fatores de Risco, Morbidade, Mortalidade

O novo coronavírus (SARS-CoV-2, do inglês *Severe Acute Respiratory Syndrome Coronavirus* -2) é o agente etiológico da COVID-19 (doença do novo coronavírus 2019), cujo surto foi declarado pandemia pela Organização Mundial da Saúde em 11 de março de 2020.^1^ Apesar de terem sido obtidos o isolamento, o sequenciamento genético e a análise estrutural do SARS-CoV-2, ainda não existem terapias especificas para COVID-19.

Os primeiros dados epidemiológicos indicam pior evolução e maior mortalidade para os pacientes com COVID-19 portadores de doenças crônicas, como cardiopatia e hipertensão arterial. O Ministério da Saúde do Brasil expandiu esse grupo de alto risco para grávidas, puérperas e mulheres após aborto.^2^

Evidências epidemiológicas anteriores sugerem fortemente que as mulheres grávidas têm um risco aumentado de doenças graves e morte por infecções virais durante pandemias, como a gripe.^3^

*Alterações fisiológicas no período gestacional não aumentam apenas suscetibilidade a infecção viral, mas também a gravidade dessa doença ( 1)* .^4 - 6^ Durante a gravidez, a resposta imune predomina através das células T-helper 2 (Th2), que protegem o feto, mas tornam a mãe mais vulnerável a infecções virais, que são combatidas de forma mais eficaz pelas células Th1.^7^

Grávidas infectadas com o vírus influenza, subtipo H1N1, e com dois outros coronavírus patogênicos, SARS-CoV (do inglês, *severe acute respiratory syndrome coronavirus* ) e MERS-CoV (do inglês, *Middle East respiratory syndrome coronavirus),* apresentaram alta morbimortalidade durante a gravidez e após o parto. Estimou-se que 90% das grávidas com essas infecções virais evoluíram para insuficiência respiratória grave com complicações obstétricas, como aborto, parto prematuro e crescimento intrauterino restrito. Em grávidas infectadas com SARS-CoV ou MERS-CoV, foi relatada mortalidade materna de até 25%, mas não houve relato de transmissão vertical transplacentária.^8^

*Não há dados de que a gravidez aumente a suscetibilidade à COVID-19.* Apesar de escassa evidência, parece que a COVID-19 durante a gravidez é menos grave do que as infecções pelo vírus influenza subtipo H1N1, SARS-CoV e MERS-CoV.

*Estudos em grávidas infectadas com o SARS-CoV-2 são limitados a pequenas séries* . Uma revisão sistemática^9^ de 108 grávidas com COVID-19 identificou tosse e febre como as principais queixas, presentes em quase 80% das mulheres, enquanto dispneia foi informada por apenas 12%. Não há relato de morte materna. Das três pacientes graves que precisaram de ventilação mecânica, duas apresentavam obesidade (índice de massa corporal > 35) como fator de morbidade.

Outro estudo^10^ avaliando 116 grávidas com pneumonia por COVID-19 concluiu que as características clínicas da pneumonia das grávidas eram semelhantes àquelas da população geral. Atualmente não há evidência de que grávidas com COVID-19 estejam mais propensas a desenvolver pneumonia grave em comparação a não grávidas. Felizmente, não houve aumento de aborto espontâneo nem de parto prematuro natural, nem ainda evidência de transmissão vertical de SARS-CoV-2.

*Transmissão perinatal de COVID-19: Devemos nos preocupar?* Dos 75 recém-nascidos de mães com COVID-19, apenas um foi positivo para o vírus e apresentou evolução clínica satisfatória com leve alteração nas enzimas hepáticas.^9^ Entretanto, alguns bebês que testaram negativo para COVID-19 apresentaram linfocitopenia e achados radiológicos de pneumonia, e um apresentou coagulação intravascular disseminada. Todos os bebês se recuperaram totalmente.^11 , 12^ Em vista disso, não se pode excluir a possibilidade de uma resposta subclínica de fetos e recém-nascidos à infecção materna, nem transmissão vertical transplacentária. Portanto, recomenda-se o monitoramento cuidadoso de recém-nascidos de mães com COVID-19.

*As grávidas com cardiopatia ou hipertensão arterial e infecção pelo SARS-CoV-2 devem ser consideradas de maior risco para mortalidade?* O documento da Sociedade Brasileira de Cardiologia para conduta em doenças cardíacas na gravidez,^13^ que inclui protocolos de cuidados, estratégias de tratamento e prevenção de complicações cardíacas durante a gravidez, contribuiu para a redução da mortalidade materna no Brasil. No entanto, enfrentamos a emergência da COVID-19, uma doença que compromete essas conquistas. A continuidade das pesquisas clinicas e novas abordagens para integrada para o subgrupo de grávidas com cardiopatia ou hipertensão afetadas pelo SARS-CoV-2 são mandatórias.

**Tabela 1:** – Impacto das alteraçôes fisiológicas cardiovasculares e respiratórias em grávidas cardiopatas com SARS-CoV-2

Desequilibrio do sistema imune materno: atenuação Th1 e dominância Th2 - Risco de infecções virais
Aumento do consumo de oxigênio: hipoventilação, apneia ou comprometimento da troca gasosa - Hipoxemia materna e fetal
Diminuição da capacidade residual funcional pulmonar materna (10%-25%) - Hipoxemia
Hiperemia e edema de vias aéreas superiores - Dificuldade na intubação endotraqueal
Aumento do Volume das mamas e necessidade de intubação e sequência rápida devido a esvaziamento gástrico retardado - Risco de aspiração
Redução da resistência vascular sistêmica - Hipotensão materna e hipoxemia
Aumento da frequência cardíaca e do volume sistólico - Insuficiência cardíaca
Ventilação Mecânica
Hiperventilação e alcallose respiratória - Vasoconstrição uterina
Hipoventilação e hipercapnia - Acidose respiratória fetal
PaO2 materna deve ser mantida ≥ 70 mmHg - Adequada oxigenação fetal
Risco aumentado de tromboemlismo
Aumento nos fatores de coagulação (V, VIII, X e von Willebrand)
Redução nos níveis de proteína S
Compressão uterina da veia cava inferior e da veia ilíaca esquerda
Trauma local da veia cava inferior e da veia ilíaca esquerda durante o parto
Após parto cesárea

A perspectiva de desfecho otimista para a combinação de gravidez e infecção pelo SARS-CoV-2 torna-se incerta em mulheres com cardiopatia ou distúrbios hipertensivos, pois essas duas condições cardíacas isoladamente representam as principais causas de mortalidade materna e fetal na gravidez.

É importante que a suspeita clínica de COVID-19 em mulheres grávidas com doença cardíaca seja descartada. Doenças cardíacas e COVID-19 têm sintomas em comum, o que pode levar a erro diagnóstico ( Tabela 2 ). Em vista disso e considerando a atual pandemia, testes para SARS-CoV-2 devem fazer parte das boas práticas de triagem para grávidas com doença cardíaca.

**Tabela 2:** – COVID-19 / Cardiopátia / Gravidez - Características e diagnóstico diferencial da triade

	Covid-19	Cardiopatia	Gravidez normal
Sintomas	Febre (>37,8 ºC), mialgia, fadiga, anorexia, dor de garganta, congestão nasal e conjuntival, tosse, dispneia, anosmia, anorexia, odinofagia, náusea, vômito, diarreia, dor abdominal.	Dispneia, palpitação, dor torácica, sincope, hemoptise, fadiga, edema de membros inferiores, ortopneia, tosse seca	Náusea, vômito, edema, dispneia, fadiga, palpitações, tontura, epistaxe, rinite gestacional, cefaleia, dor abdominal
Aparecimento dos sintomas e idade gestacional	Qualquer idade gestacional ou puerpério	Em geral no 2 e 3 trimestre e puerpério	Qualquer idade gestacional
História	Sem cardiopatia prévia	Cardiopatia prévia	Sem cardiopatia prévia
Exames laboratoriais	RT-PCR swab nasofaringeo positivo para COVID-19 Linfocitopenia Aminotransferases (ALT/AST): elevadas ureia/creatinina alteradas Dimero d: elevado	Peptídeo natriurético tipo B: niveis altos	Dimero d: normal ou levemente elevado
Exames de imagem	Ecocardiograma: normal Raio-X de tórax: com ou sem alteração Tomografia de tórax: opacidade em “vidro fosco”	Ecocardiograma: lesão cardíaca estrutural Alteração de raio- X/tomografia de torax: cardiomegalia e/ou congestão pulmonar	Ecocardiograma: normal Raio-X de tórax: normal

*RT-PCR: em inglês: reverse transcription polymerase chain reaction assay; ALT: alanina aminotransferase; AST: aspartato aminotransferase.*

*As Alterações fisiológicas no sistema cardiorrespiratório devidas à gravidez não aumentam a suscetibilidade à infecção pelo vírus, mas podem piorar o desfecho materno*^4 - 6^ . As alterações respiratórias na gravidez levam a redução da capacidade pulmonar total e da complacência torácica no final da gestação. Além disso a hipóxia materna decorrente da hipoventilação e do comprometimento nas trocas gasosas reduz a oferta de oxigênio para o feto, e consequentemente, morte intra-uterina. Nesse contexto, a pneumonia da COVID-19 progride rapidamente de consolidação pulmonar focal para difusa bilateral, predispondo a grave insuficiência respiratória hipoxêmica.

*COVID-19 pode levar a injúria cardíaca por múltiplos mecanismos, resultando em resposta inflamatória extrema com lesão endotelial e miocardite.*^14^ Na gravidez e no período pós-parto, insuficiência cardíaca aguda deve ser considerada em algumas circunstâncias, como cardiomiopatia periparto, miocardite viral e edema pulmonar não cardiogênico. Edema pulmonar é também visto em grávidas saudáveis em decorrência de importantes alterações no volume intravascular durante o trabalho de parto e após o parto. Da mesma forma, alterações hemodinâmicas na gestação causam aumento no gradiente da válvula mitral estenótica e pode levar a congestão pulmonar. A cardiopatia congênita cianótica, as lesões obstrutivas do lado esquerdo do coração ou grave disfunção ventricular sistólica apresentam maior risco de complicações cardíacas em mulheres grávidas. A queda da resistência vascular sistêmica piora a hipoxemia em mulheres grávidas com hipertensão pulmonar e com tetralogia de Fallot não corrigida.

*Coagulopatia sistêmica é um aspecto crítico de morbimortalidade na COVID-19.*^14^ O estado de hipercoagulabilidade da gravidez eleva o risco de tromboembolismo em mulheres cardiopatas. Nesse cenário, a combinação de COVID-19 e prótese valvular mecânica ou fibrilação atrial na doença valvar reumática aumenta o risco de eventos tromboembólicos em mulheres grávidas. Vale ressaltar que, a cada trimestre da gestação, os níveis de dímero-D aumentam, fato que deve ser considerado na interpretação para diagnóstico de tromboembolismo pulmonar.

*Inflamação sistêmica e coagulopatia na COVID-19 elevam o risco de ruptura da placa aterosclerótica e infarto agudo do miocárdio.*^14^ A significativa implicação de infecção pelo SARS-CoV-2 para o sistema cardiovascular é evidenciada por injúria miocárdica aguda (altos níveis de troponina I ultrassensível e/ou novas anormalidades no ECG/ecocardiograma), arritmias cardíacas complexas e parada cardíaca. Durante a gravidez, síndrome coronariana aguda não é comum. Entretanto, infecções, em especial no pós-parto, são fatores de risco para infarto do miocárdio. As causas mais frequentes de infarto do miocárdio na gravidez são dissecção espontânea de artéria coronária, aterosclerose, trombose coronariana e artérias normais na angiografia com comprometimento da microcirculação coronariana.

*Estudos recentes mostraram que a enzima de conversão da angiotensina 2 (ECA2) é um receptor funcional de SARS-CoV-2.*^15^ O sistema renina-angiotensina é o principal responsável pela regulação da pressão arterial e a ECA2 tem papel crítico no controle da fisiologia cardiovascular em grávidas. Angiotensina-(1-7) é significativamente elevada em grávidas saudáveis quando comparadas a não grávidas. Na pré-eclâmpsia, os níveis plasmáticos de angiotensina (1-7) são reduzidos e a angiotensina II plasmática é consistentemente elevada, o que contribui para o desenvolvimento de hipertensão nessas gestantes. Além disso, grávidas com hipertensão crônica apresentam maior risco de pré-eclâmpsia ou síndrome HELLP. Contudo, a relação entre regulação positiva de ECA2 e SARS-CoV-2 na gravidez requer estudos adicionais.

Por fim, atualmente, não há dados sobre o desfecho da gravidez em pacientes com cardiopatia ou hipertensão arterial e COVID-19. Entretanto, essas pacientes têm que ser consideradas um grupo de alto risco.

Devido à falta de terapêutica específica e de vacina para COVID-19, precisamos estar preparados para prevenir e tratar complicações cardiovasculares na gestação.^13^ Cuidado integrado e multidisciplinar deve visar à otimização da terapia, à orientação das pacientes quanto aos riscos da COVID-19 e ao seu tratamento em uma eventual infecção por SARS-CoV-2.

As graves consequências da COVID-19 somadas às complicações de grávidas com cardiopatia ou hipertensão arterial podem determinar pior desfecho materno e prognóstico incerto.
