# Supplementary material for: COVID-19: Um Novo Desafio para a Cardiopatia na Gravidez
Source: Arq Bras Cardiol. 2020 Jul 28;115(1):1–4. [Article in Portuguese] doi: 10.36660/abc.20200511 (PMC8384333; doi:10.36660/abc.20200511)
Supplement: Translation

# Translation: COVID-19: A New Challenge in Pregnancy and Heart Disease

**Authors:** Walkiria Samuel Avila, Regina Coeli de Carvalho

**Affiliations:** 1 Brasil Universidade de São Paulo Instituto do Coração, São Paulo, SP - Brasil; 2 Brazil Hospital Geral de Fortaleza, secretária de Saúde do Estado do Ceará (SESA), Fortaleza, CE - Brazil

**Keywords:** Coronavirus, COVID19, Pandemics, Pregnancy, Pregnancy, high-risk, Severe Acute Respiratory Syndrome, Pneumonia, Hypertension/prevention and control, Risk Factors, Morbidity, Mortality

The outbreak of coronavirus disease 2019 (COVID-19), caused by the severe acute respiratory syndrome coronavirus 2 (SARS-CoV-2), was declared a pandemic by the World Health Organization on March 11, 2020.^1^ Although SARS-CoV-2 isolation, gene sequencing, and structural analysis have been completed,therapies for COVID-19.

The first epidemiological data have pointed to a worse outcomes and higher mortality of COVID-19 in patients with chronic diseases, such as heart disease and arterial hypertension. Subsequently, the Brazilian Health Ministry included in the high-risk group pregnant, puerperal and post-abortion women.^2^

Previous epidemiological evidence strongly suggests that pregnant women have an increased risk of serious illness and death from viral infections during pandemics, such as influenza.^3^

*Physiological changes in pregnant women not only increase susceptibility to the viral infection but also increase the severity of this disease* ( *Table 1 )* .^4 - 6^ During pregnancy, the immune response predominates through T-helper 2 (Th2) cells, which protect the fetus but make the mother more vulnerable to viral infections, which are more effectively fought by Th1 cells.^7^

**Table 1:** – Impact of physiological changes of cardiovascular and respiratory systems of pregnancy in women with cardiac disease with SARS-CoV-2

Downregulation of maternal immune system - Risk to Severe Respiratory Disease
Oxygen consumption - hypoventilation, apnea or impaired gas exchange - Hypoxemia
Decreased Functional Residual Capacity - (10 a 25%) - Hypoxemia
Hyperemia and edema of the upper airway - Challenges to endotracheal intubation
Increased breast volume and the need for crush induction due to impaired gastric emptying - Risk of aspiration
Decreased systemic vascular resistance - Hypotension, Hypoxemia
Incresead heart rate and stroke volume - Heart failure
Mechanical ventilation carefulness
Hyperventilation and alkalosis - Uterine vasoconstriction
Hypoventilation and hypercapnia - Fetal respiratory acidosis
Matenal PaO_2_should be kept greater than 70 mmHg for adequate fetal oxigenation
Increase thromboembolic risks
increase in coagulation factors (V, VIII, X, and von Willebrand factor)
Fall in protein S levels
Uterine compression of the inferior vena cava and the left iliac vein
Local trauma to pelvic veins during delivery
Pospartum period of cesarean section

Pregnant women infected with the H1N1 influenza virus and two other pathogenic coronaviruses [severe acute respiratory syndrome coronavirus (SARS-CoV) and Middle East respiratory syndrome coronavirus (MERS-CoV)] had high morbidity and mortality during pregnancy and the postpartum period. It was estimate that 90% of pregnant women with these viral infections progressed to severe respiratory failure, with obstetric complications, such as abortion, premature birth, and intrauterine growth restriction. Maternal mortality in pregnant women infected with SARS-CoV or MERS-CoV has been reported to reach 25%, and no transplacental vertical transmission has been recorded.^8^

*There are no data to inform whether pregnancy increases susceptibility to COVID-19.* The evidence is still scarce, but COVID-19 during pregnancy appears to be less severe than infections by H1N1 influenza virus, SARS-CoV, and MERS-CoV.

*Studies in pregnant women infected with SARS-CoV-2 are limited to small series* . A systematic review^9^ of 108 pregnant women with COVID-19 has shown cough and fever as their main complaints, present in almost 80% of the women, while only 12% had dyspnea. No maternal death has been reported. Of the three critical patients who required mechanical ventilation, two had obesity (body mass index > 35) as a morbidity factor.

Another study^10^ assessing 116 pregnant women with COVID-19 pneumonia has concluded that clinical pneumonia characteristics of pregnant women were similar to those of the general population. Currently, there is no evidence that pregnant women with COVID-19 are more prone to develop severe pneumonia as compared to nonpregnant ones. Fortunately, there was neither increase in spontaneous abortion or in natural preterm birth, nor evidence of vertical transmission of SARS-CoV-2.

*Perinatal transmission of COVID-19: Should we be concerned?* Of 75 newborns of mothers with COVID-19, only one tested positive for the virus and had a satisfactory clinical outcome with mild changes in liver enzymes.^9^ However, some babies testing negative for COVID-19 developed lymphocytopenia and radiological findings of pneumonia, and one had disseminated intravascular coagulation. All babies had a full recovery.^11 , 12^ Based on these findings, we can exclude neither the possibility of subclinical response from fetuses and newborns to the maternal infection, nor transplacental vertical transmission. Thus, close monitoring of newborns of mothers with COVID-19 is recommended.

*Should pregnant women with heart disease or arterial hypertension and SARS-CoV-2 infection be considered at greater mortality risk?* The Brazilian Society of Cardiology Statement for the Management of Cardiac Diseases in Pregnancy,^13^ which includes healthcare protocols, treatment, and prevention strategies for heart complications during pregnancy, has contributed to a reduction in maternal mortality in Brazil. However, we are facing the emergence of COVID-19, a disease that shakes that once improved situation. New clinical research and an integrated approach to the subgroup of pregnant women with heart disease or hypertension affected by SARS-CoV-2 are mandatory.

The optimistic perspective on the outcomes of the association of pregnancy and SARS-CoV-2 infection becomes uncertain in women with heart disease or hypertensive disorders, because these two heart conditions alone represent the main causes of maternal and fetal mortality during pregnancy.

It is important that clinical suspicion of COVID-19 in pregnant women with heart disease MUST be ruled out. Heart diseases and COVID-19 have symptoms in common, which can lead to a misdiagnosis ( Table 2 ). In view of this and considering the current pandemic, tests for SARS-CoV-2 should be included in the good practice screening for pregnant women with heart disease.

**Table 2:** – COVID-19 / Heart Disease / Pregnancy - Features of trinity and Differential Diagnosis

	Covid-19	Cardiopathy	Normal Pregnancy
Symptoms	Fever (>37,8 ºC), myalgia, fatigue, anorexia, sore throat, nasal and conjuctival congestions, cough, Dyspnea, anosmia, anorexia, odynophagia, nausea, vomiting, diarrhea, abdominal pain	Dyspnea, palpitations, chest pain, syncope, hemoptysis, fadigue, lower limb edema, orthopnea, dry cough.	Náusea, vomiting, edema / dyspnea / fatigue, palpitations, dizziness, epistaxis, gestational rhinitis, headache and abdominal pain
Time of symptoms and age gestational	Any of gestational age	2^nd^and 3^rd^trimester	Any gestational age
History	Without previous heart disease	Previous heart disease	Without previous heart disease
Laboratory	COVID-19 Positive RT-PCR nasopharygeal Lymphocytopenia Increased aminotransferase- Alt/AST ureia / creatinine D-dimer -increased	High level of B-type natriuretic peptide BNP	D - dimer normal or slight increase
Imaging Exams	Normal echocardiogram Chest X-ray alterede or not Chest tomography - ground glass opacities image	Echocardiogram - structural cardiac lesion Chest X ray / Tomography altered: Cardiomegaly and / or pulmonary congestion	Normal echocardiogram Normal Chest-X ray

*RT-PCR: reverse transcription polymerase chain reaction assay; ALT: alanine aminotransferase; AST: aspartate aminotransferase.*

*Physiological changes in the cardiorespiratory system due to pregnancy do not increase susceptibility to infection by the virus but can induce worse maternal outcome*^4 - 6^ ( Table 1 ). Respiratory changes in pregnancy result in decreased total lung capacity and chest compliance at the end of pregnancy. In addition, it is reasonable to consider that maternal hypoxia resulting from hypoventilation and impaired gas exchange reduces the offer of oxygen to the fetus, consequently, intra-uterine death. In this context, COVID-19 pneumonia progresses rapidly from focal to bilateral diffuse pulmonary consolidation and more readily predisposes to severe hypoxemic respiratory failure.

*COVID-19 can result in cardiac injury by multiple mechanisms, resulting in an extreme inflammatory response with endothelial injury and myocarditis.*^14^ During pregnancy and the postpartum period, acute heart failure should be considered in some circumstances, such as peripartum cardiomyopathy, viral myocarditis, and noncardiogenic pulmonary edema. Pulmonary edema is also seen in healthy pregnant women, as a consequence of major changes in intravascular volume during labor and after delivery. Likewise, hemodynamic changes in pregnancy cause an increase in the gradient across the stenotic mitral valve and could lead to pulmonary congestion. Congenital cyanotic cardiopathy, obstructive injuries of the left side of the heart or serious systemic ventricular dysfunction present a greater risk of cardiac complications in pregnant women. The fall in systemic vascular resistance worsens hypoxemia in pregnant women with pulmonary hypertension and with uncorrected tetralogy of Fallot

*Systemic coagulopathy is a critical aspect of morbidity and mortality in COVID-19.*^14^ The hypercoagulable state ( Table 1 ) of pregnancy increases the risk of thromboembolism in women with heart disease. In this scenario, the combination of COVID-19 and mechanical valve prosthesis or atrial fibrillation in rheumatic valve disease increases the risk of thromboembolic events in pregnant women. It is worth noting that, as D-dimer levels increase as pregnancy progresses, it is not a good marker for the diagnosis of thromboembolism in pregnancy. Despite radiation exposure, chest computed tomography and complementary angiography should be indicated in pregnant women with heart disease and COVID-19, when pulmonary thromboembolism is suspected.

*Systemic inflammation and coagulopathy in COVID-19 increase the risk of atherosclerotic plaque rupture and acute myocardial infarction.*^14^ The significant implication of SARS-CoV-2 infection for the cardiovascular system is evidenced by acute myocardial injury (high levels of highly sensitive troponin I and/or new ECG/echocardiogram abnormalities), complex cardiac arrhythmias, and cardiac arrest. During pregnancy, acute coronary syndromes are not common. However, infections, especially in the postpartum period, are a risk factor for myocardial infarction. The most frequent causes of myocardial infarction during pregnancy are spontaneous coronary artery dissection, atherosclerosis, coronary thrombosis, and normal arteries on angiography with impaired coronary microcirculation

*According to recent studies, angiotensin-converting enzyme 2 (ACE2) is a functional receptor of SARS-CoV-2.*^15^ The renin-angiotensin system is a key player in blood pressure regulation, and ACE2 plays a critical role in cardiovascular physiology control in pregnant women. Angiotensin-(1-7) is significantly elevated in healthy pregnant women as compared to nonpregnant ones. In preeclampsia, plasma angiotensin levels (1-7) are reduced and plasma angiotensin II is consistently elevated, which contributes to the development of hypertension in these pregnant women. Moreover, pregnant women with chronic hypertension are at risk for preeclampsia or HELLP syndrome. Therefore, the relationship between positive regulation of ACE2 and SARS-CoV-2 in pregnancy requires further studies.

Finally, there are currently no data available on the outcome of pregnancy in patients with heart disease or arterial hypertension and COVID-19. However, those patients must be considered a high-risk group.

In view of the lack of specific therapy and vaccine for COVID-19, we have to be prepared to prevent and treat cardiovascular complications during pregnancy.^13^ Integrated and multidisciplinary care should be aimed at optimizing therapy, guiding patients on the risks of COVID-19, and treating them in an occasional infection by SARS-CoV-2.

The severe consequences of COVID-19 compounded by the possible complications experienced by pregnant women with heart disease or arterial hypertension could result in poor maternal outcome and uncertain prognosis.
